# Re-visiting the six-item Stanford presenteeism scale (SPS-6) and its psychometric properties

**DOI:** 10.3389/fpsyg.2023.1251357

**Published:** 2023-09-28

**Authors:** Frank Bezzina, Agnieszka Zielińska, Vincent Cassar

**Affiliations:** ^1^Department of Business and Enterprise Management, Faculty of Economics, Management and Accountancy, University of Malta, Msida, Malta; ^2^Department of Management, University of Bielsko-Biala, Bielsko-Biała, Poland

**Keywords:** presenteeism, Stanford presenteeism scale, psychometric properties, occupational well-being, health-related productivity, JD-R model

## Abstract

Presenteeism has often been considered as the correlate of absenteeism and associated to productivity loss. This study sought to re-examine the psychometric properties of the 6-item Stanford Presenteeism Scale (SPS-6), a popular measure which has been translated in a number of languages. The study adopted a cross-sectional design with 268 participants aged 18 - 65 working in a multinational IT company with headquarters based in Poland. The respondents participated willingly in an online questionnaire on a presenteeism health-related productivity measure (SPS-6), job resources (peer support), job demands (work-to-family conflict), engagement and burnout. Their responses were subjected to statistical analyses. Confirmatory Factor Analysis revealed that the SPS-6 is better represented by two singular and independent components, namely completing work and avoiding distractions, rather than an aggregated measure of health-related productivity. In fact, the aggregated measure had convergent and discriminant validity issues. We also assessed, via Structural Equation Modeling (SEM), the explanatory role of the SPS-6 within the wider well-being discourse by subjecting its’ factors as outcomes using the JD-R framework. Here, burnout was better at explaining its relationship to avoiding distractions and completing work compared to engagement, while avoiding distractions was more dominant than completing work in explaining indirect pathways. Given the convergent and discriminant validity of its two-dimensional measures, we argue that the SPS-6 is a better assessment of health-related productivity in the light of presenteeism when keeping both components separate rather than adding the scores from both dimensions to provide a global score as has been the practice so far. In addition, the SEM findings suggest that both SPS-6 components may require different theoretical explanations. This study supports a growing chorus of scholars who argue the need to look deeper into the presenteeism phenomenon, not least its measures.

## Introduction

The notion of presenteeism, generally defined as going to work despite feeling unhealthy or experiencing sickness (Aronsson et al., 2000; Evans, 2004; Dew et al., 2005; Johns, 2010; Côté et al., 2021), is an important topic because it has both health and economic implications. Since its inception (Cooper, 1996), the phenomenon has been actively investigated and debated (Johns, 2010) and has been closely associated with absenteeism (Gosselin et al., 2013; Ruhle and Süß, 2020). Past studies have examined both the antecedents of presenteeism (e.g., Aronsson and Gustafsson, 2005; Lohaus and Habermann, 2019) and its consequences including productivity (e.g., Collins et al., 2005) and health related symptoms (e.g., Gustafsson and Marklund, 2014). While a search in the Web of Science will extract less published articles compared to absenteeism over the last 22 years (2,120 compared to 7,500), presenteeism is known to have a number of health and financial costs to organizations (Gosselin et al., 2013; Karanika-Murray and Biron, 2020). For example, in the US alone, presenteeism is thought to cost companies over 150 billion dollars (Hemp, 2004). More recently, Brunner et al. (2019) estimated that productivity losses amount to approximately 205 Euro per person per month although their estimation also accounts for the cost of absenteeism. Presenteeism may be considered as more significantly costly due to the fact that presenteeism is not always so obvious or visible. Indeed, it requires a period of time and serious damages for act identification (Ruhle and Süß, 2020).

In spite of this growing knowledge, theoretical, conceptual and measurement, issues still remain (Lohaus and Habermann, 2019) and more recently Patel et al. (2023) have re-sounded the alarms about the many lacunae and gaps that are still of concern. It is in some ways quite surprising that after almost three decades since the inception of the construct, there are still unresolved issues. These mainly arise out of the need to capture a complex and multifaceted phenomenon within a simpler conceptual framework with some scholars viewing the phenomenon from a wide array of perspectives in an attempt to decouple the ‘cause’ from the ‘effect’ (Cooper and Lu, 2016). These range from the state or act of deciding to go to work while feeling ill (Aronsson and Gustafsson, 2005) to understanding presenteeism as a function of the state of one’s illness to still attend work (Hansen and Andersen, 2008) to conceptualizing presenteeism as represented or captured by the aftermath of this dynamic process leading to loss of productivity (Collins et al., 2005; Li et al., 2019). Adding to this conceptual challenge is the fact that a number of measures have been developed claiming to assess presenteeism (Ospina et al., 2015). Indeed, while the construct still requires various clarifications (Gosselin et al., 2013; Patel et al., 2023) some of the measures still lack criterion validity (Lohaus and Habermann, 2019). Measures include the Health and Work Performance Questionnaire (Kessler et al., 2004), the Health and Work Questionnaire (Shikiar et al., 2004), the Work Limitations Questionnaire (Lerner et al., 2001), the Workplace Attendance-Behavior Legitimacy Scale (Ruhle and Breitsohl, 2023) and the Stanford Presenteeism Scale (SPS) (Koopman et al., 2002) which is the subject of our paper.

The reasons for assessing this measurement of presenteeism are various. First, the SPS-6 represents a typical and popular measure of presenteeism (like most of self-report measures in this field) with its focus on productivity loss as a function of one’s health status. Second, the SPS-6 is a widely used measure that has been translated into various languages. These include Portuguese (Laranjeira, 2013), Brazilian-Portuguese (Frauendorf et al., 2014), Italian (Cicolini et al., 2016), Dutch (Hutting et al., 2014), Spanish (Baldonedo-Mosteiro et al., 2020), and Persian (Abdi et al., 2021). Third, it attempts to capture a wide spectrum of the phenomenon. The SPS-6 is a multi-item measure that encapsulates the phenomenon of presenteeism from a cognitive, affective and behavioral aspect encompassing the non-formal hours of work and worksite. Fourth, it offers a good opportunity to explore some of the underlying inconsistencies and gaps that also reflect the on-going discussion on presenteeism measurement and hence supports its theoretical and measurement development (Lohaus and Habermann, 2019). Fifth, the measure has increasingly been associated to productivity-*loss* rather than the act of presenteeism itself (Collins et al., 2005; Li et al., 2019) although, in line with the notion that the concept is a complex construct (Cartwright and Cooper, 2014) and the way the SPS-6 has been handled, its mere connotation to ‘loss’ may be slightly incorrect. We posit that construct clarity and theory are two sides of the same coin and require clarity and justification (Suddaby, 2010) which therefore reflects the purpose of the measure. Therefore, the objectives of the study are threefold. First, to re-evaluate the factor structure of the SPS-6 and test for its convergent and discriminant validity; second, to examine whether the SPS-6 is better represented by two singular and independent components or an aggregated measure of presenteeism as recommended by its proponents (i.e., Koopman et al., 2002); and thirdly, to investigate how presenteeism behaves within a framework of occupational well-being.

### Conceptualizing presenteeism and its association to absenteeism

A number of papers address presenteeism in conjunction with absenteeism. Some looked into the underlying factors of both presenteeism and absenteeism (Prater and Smith, 2011; Gosselin et al., 2013) while a more recent study discussed sickness-related (non)attendance behavior in view of both presenteeism and absenteeism (Ruhle and Süß, 2020). Indeed, there is a degree of phenomenal affinity between presenteeism and absenteeism and it has become recognized that the concept is best examined by taking stock of the knowledge gained about absenteeism (Gosselin et al., 2013). Brunner et al. (2019), in their analysis, weighed the monetary and economic benefits of resources and the constraints of stress on work attendance behavior of Swiss employees contemplating either to stay at home (absenteeism) or deciding to still go to work in spite of their ill-state (presenteeism). However, while presenteeism is a practice of going to work sick or injured, which is often assumed to result in reduced productivity, absenteeism is defined as regular (physical) absence from work (Prater and Smith, 2011). It has been argued that the main difference between these two phenomena is that while absenteeism is at the top of the productivity loss iceberg, presenteeism is at the underwater part of this iceberg (Johns, 2016). In general, presenteeism is considered as the antithesis of absenteeism (Prater and Smith, 2011), since in the first instance workers may attend work with harmful consequences on both their health and productivity; in the second they may not manifest such effects except for the lack of productivity owing to their absence. Hence, both phenomena may, at face value, be conceptualized from a productivity loss perspective. However, although the productivity loss from absenteeism can be calculated, presenteeism represents an indirect cost as it is more invisible (Hemp, 2004) and presenteeism is more insidious and harder to estimate than absenteeism in terms of its real impact on productivity as its manifestation may result in other collateral impacts (Brooks et al., 2010). In fact, while presenteeism costs up to 180 billion dollars per year, the costs of absenteeism are actually lower and calculated at 118 billion dollars per year (Weaver, 2010). In the meantime, others have argued that presenteeism may *lead* to absenteeism, and hence presenteeism can be classified as a precursor to absenteeism and other collateral risks (Gustafsson and Marklund, 2011). In this case, the possible outcome from illness-related presenteeism concerns productivity loss, quality reduction and lower speed of work. Apart from these, there is a decline in health conditions; hence presenteeism today might trigger absenteeism tomorrow, since employees are simply not capable of attending work. Unsurprisingly, a relation between these two behaviors has been found (Gosselin et al., 2013; Garrow, 2016), and it is possible that sick leave results in a reduced salary, which pressures people to stay at work and be affected by presenteeism. Therefore, while presenteeism and absenteeism are different phenomena, they are closely related to each other.

### Presenteeism and productivity loss?

‘Presenteeism’ as a construct still struggles to be properly defined (Patel et al., 2023) although a number of authors seem to agree that this is associated with the conscious desire and personal decision to attend work while feeling or being ill (Johns, 2010). At the heart of this definition is the notion of personal decision-making to attend work and that presenteeism is an adaptive mechanism intended to alleviate the potential negative effects of ‘letting go’ by remaining at work. This conceptual perspective of presenteeism highlights the notion that people are present at work irrespective of the severity of the ill-health but being adaptive also implies that when the ailment is severe enough to preclude productivity, then the notion of presenteeism ceases to exist (Karanika-Murray and Biron, 2020). Hence, this conceptualization implies that presenteeism has a negative undertone (the person is sick or feeling sick) but it also contains a positive element in that during the act of presenteeism the individual can still engage in work and this may indeed have the possibility of re-establishing a state of wellness in the person as work has meaningful and beneficial recovery effects. In fact, scholars have refrained from just assuming the term to convey negative connotations and in this sense, the construct departs from the notion of absenteeism. This thesis is also supported empirically. For instance, it has been found that presenteeism was beneficial for performance evaluations especially under high work demands (Wang et al., 2022). In addition, the general connotation that presenteeism is a precursor to direct productivity loss and performance (Johns, 2010) has not always been upheld by research and studies have generally found a weak or negative relationship between presenteeism and performance (Lu et al., 2013; Collins et al., 2018). Hence, presenteeism should best be viewed from a dual perspective: it is both the tendency for people to come to work while feeling sick (negative connotation) and the propensity to consider the act of coming to work as an adaptive mechanism to overcome the severe consequences of being sick (positive connotation). Indeed, this perspective has been propagated by a number of authors (Ruhle et al., 2020; Biron et al., 2022). Nevertheless, while the notion of a positive side to presenteeism has been upheld in its antecedent form, the same consideration does not seem to have been given to its outcomes. Some researchers have called out for exploring how presenteeism may lead to good performance evaluations (Wang et al., 2022), given its relevance may be attributed to both positive and negative outcomes even though most definitions carry negative connotations and disregard the possible positive effects of presenteeism (Patel et al., 2023). We evaluate this possibility using the SPS-6 in line with other studies who have used it conveniently as a health-related productivity ‘loss’ measure (Li et al., 2019).

### The Stanford presenteeism scale and study objectives

Like many presenteeism self-report measures, the Stanford Presenteeism Scale (SPS) is an employee-based measurement method (Ospina et al., 2015). The original aim of this scale was to measure the extent of presenteeism manifested by a typical employee although some would argue otherwise. There are three versions of the Stanford presenteeism scale: SPS-32, SPS-13 and SPS-6. The SPS-32 was created “to reflect various cognitive, emotional, and behavioral aspects of accomplishing work, despite possible health problems” (Koopman et al., 2002, p. 15). It contains 32 items and has two dimensions: ‘work focus’ and ‘psychological focus’ (Lynch and Riedel, 2001). On the other hand, the SPS-6 was developed since it was argued that the SPS-32 was too long to be used effectively in practice (Koopman et al., 2002). Presenteeism was defined as one’s “ability to concentrate on and accomplish tasks despite health problem(s)” (Koopman et al., 2002, p. 17). The SPS-6 has six items and exploratory factor analysis extracted two dimensions: ‘completing work’ (generally reflecting the motivation of achieving task goals in spite of the ailment such as ‘*Despite having my (health problem) I was able to finish hard tasks in my work*’) and ‘avoiding distractions’ (generally reflecting the tendency to manage the symptoms of the ailment while at work such as *‘I felt hopeless about finishing certain work tasks, due to my health problem’*). While they identified two dimensions of presenteeism, for some atheoretical reason they recommended the SPS-6 total scores by first reverse scoring the ‘avoiding distractions’ measures and then aggregating them into what they termed as the SPS-6 total score. To this end, it has been specified that the SPS-6 total score measures the impact of an employee’s perceived ability to concentrate during work activities distracted by health variables and pain (Roy, 2011). Clearly there was no attempt to determine why the two factor scores were added up especially when presenteeism was construed as ‘negative’. For instance, Li et al. (2019) summed up both dimensions to elicit a measure of health-related productivity loss. This lacuna was clearly emphasized when it was noted that a number of definitions of presenteeism in its broader sense lacked any scientific relevance and in fact states “that although all of the definitions pertain to being physically present at work, they differ to a greater or lesser extent from each other, occasioning potential confusion” (Johns, 2010, p. 520). Failing to understand the underlying reasons for measurements used to currently assess the outcomes of presenteeism enhances this confusion. This has been amplified by several scholars who strongly posit the need to position constructs within their theoretical construction (Suddaby, 2010).

The main aim of the current study is to clarify the SPS-6 measure within the theoretical underpinnings of its use in presenteeism research (Koopman et al., 2002). To achieve this, the study reviews a number of psychometric properties that underlie the SPS-6. First is to ascertain the internal psychometric properties of the SPS-6. Its psychometric qualities are debated by various authors. For instance, it has been stated that currently there is a lack of supporting evidence on the good psychometric qualities of the scale (Roy, 2011) while others stated the exact opposite (Hutting et al., 2013). The two most popular measures of reliability when assessing the psychometric properties of instrument measures are test–retest reliability and internal consistency reliability. Test–retest reliability assesses temporal stability and generally a space of 2 weeks is allowed between test and retest. In the case of SPS-6, Koopman et al. (2002) argued that presenteeism is not consistent over time and hence did not assess test–retest reliability. Hutting et al. (2014), however, argued that it is possible to assess test-rest reliability for a group over an intervention and in fact provided evidence of test–retest reliability (Spearman rho = 0.82, *p* < 0.01). On the other hand, internal consistency reliability refers to the “inter-relatedness of the items” in a concept or construct (Tavakol and Dennick, 2011, p. 53). It has been shown that the SPS-6 has good internal consistency reliability since Cronbach alphas ranging from 0.72 to 0.89 have been reported (Koopman et al., 2002). However, Cronbach’s alpha alone does not provide evidence of uni-dimensionality (Bezzina and Saunders, 2014). In fact, there is evidence of the underlying two-factor structure of the SPS-6 via Exploratory Factor Analysis (EFA) (Koopman et al., 2002) and that the first-order two factor model fits the data well via Confirmatory Factor Analysis (CFA) (Abdi et al., 2021). Empirical studies have reported evidence of convergent validity by establishing a significant association between the SPS-6 and some other presenteeism measures such as the Work Ability Inventory (Abdi et al., 2021), or the percentage of time workers were productive despite health problems (Koopman et al., 2002). At the same time, discriminant validity was established by providing an association with other constructs such as work ability (Abdi et al., 2021), job stress and job satisfaction (Koopman et al., 2002; Hutting et al., 2014) or made reference to external validity by establishing an association between their native version of SPS-6 and the Perceived Stress Scale or PSS-10 (Cicolini et al., 2016). However, these assessments provided evidence of criterion/concurrent validity and not convergent/discriminant validity, clearly a deviation from accepted norms and conventions (Saunders and Bezzina, 2015). Convergent validity refers to the extent to which measures of a specific construct share a high proportion of common variance (Hair et al., 2010), and hence with convergent validity issues, it means that the latent factor is not well explained by its observed variables (Gaskin, 2022). Discriminant validity refers to the extent to which “the construct measures something unique and captures some phenomenon other constructs do not” (Hair et al., 2010, p. 710) and hence, with discriminant validity issues it means that the latent factor is better explained by other variables/factors than its own observed variables (Gaskin, 2022). As a result, establishing convergent and discriminant validity is an absolutely necessary component of any CFA (Cassar et al., 2020a; Gaskin, 2022; Cheung et al., 2023). It should be established before the testing of a hypothesized model and when this issue is not addressed, “the interpretation of the whole model can be misleading or useless” (Ab Hamit et al., 2017, p. 1). While many researchers report Cronbach’s alpha and factor analysis results as evidence of the quality of their measurement scales, these are typically inadequate and sometimes inappropriate (Fornell and Larcker, 1981; Cheung et al., 2023). It is generally acknowledged that useful measures for establishing convergent validity are the Composite Reliability (CR), and the Average Variance Extracted (AVE), while the Maximum Shared Variance (MSV), the Average Shared Variance (ASV) and the inter-item correlation are useful measures to establish discriminant validity (Hair et al., 2010; Gaskin, 2022). We are not aware of studies that examined the convergent and discriminant validities of the SPS-6 by using these measures. This reflects an important gap in the research on the psychometric properties of the SPS-6 and emerges as the first objective that will be addressed in this study. Our first research question is:

### Do the two dimensions of the SPS-6 (i.e., ‘completing work’ and ‘avoiding distractions’) demonstrate adequate convergent and discriminant validity?

The proponents of the SPS-6 (Koopman et al., 2002) argued that the two subscales of the SPS-6 should not be considered separately, but rather one should use the aggregated scale ranging from 6 to 30 even though their study, as well as the translated versions, confirmed the two-factor structure of the SPS-6: ‘Completing Work’ and ‘Avoiding Distractions’ (Koopman et al., 2002; Hutting et al., 2014; Cicolini et al., 2016; Baldonedo-Mosteiro et al., 2020). In such cases, one would consider conducting a second order CFA before aggregating factor scores. A second order CFA tests the assumption that the correlations among a set of first-order factors are accounted for by a single higher order factor, such that “the construct consists of a single broader dimension” (Brown, 2015, p. 288). There are various advantages of having a second order factor. For instance, it has been argued that a higher order factor provides a more elegant representation of hierarchical constructs and that “composite scores of first-order factors will be difficult to assemble and meaningfully describe and then employ in substantive hypotheses testing” (Koufteros et al., 2009, p. 635). Additionally, a higher-order factor provides a more parsimonious model (since fewer degrees of freedom are consumed) and performs better on goodness-of-fit indices that reflect parsimony (Hair et al., 2010). By reviewing second-order CFA output, particularly by assessing the strength of the factor loadings in the model as well as goodness-of-fit indices, we can determine whether or not the second-order factor may be producing the associations among the first order SPS-6 factors (Byrne, 2001). Constructing a second-order CFA model will also enable us to assess the convergent validity and the discriminant validity of the aggregated measure of the SPS-6. This reflects another important gap in the research on the psychometric properties of the SPS-6 that will be addressed in this study. Hence, our second research question is:

### Is there sufficient justification to use an aggregated measure of presenteeism or is it better to use two singular and independent components?

In the lack of statistical support for an aggregated measure of the SPS-6, this study will investigate how the two separate dimensions behave in more complex relationships. More specifically, we will be using the Job Demands-Resources model (JD-R) (Bakker and Demerouti, 2007) to investigate the concurrent validity of the two dimensions. The JD-R model became recognised as a leading occupational stress model (Cassar et al., 2020b) where working conditions are bifurcated into job demands (i.e., physical or emotional stressors in one’s role and the reasons for the health impairment process) and job resources (i.e., job positives - the physical, social and organizational factors that help one to achieve goals and manage stress). The model states that when job demands are high and job resources are low, burnout increases leading to undesirable work outcomes. Conversely, increased job resources can offset the effects of high job demands leading to enhanced engagement and therefore more positive work outcomes (Demerouti and Bakker, 2011). In view of the dual-nature of presenteeism, it is plausible to postulate that different aspects of health-related productivity outcomes may be triggered differently by demands and resources rather than merely productivity-loss. Assuming SPS-6 is best conceptualized as representing a measure of the aftermath of the act of presenteeism and assuming that the two dimensions have a life of their own, one may also consider that rather than referring to productivity-loss, one can also make reference to productivity-maintenance (e.g., Li et al., 2019).

We extend this view to postulate that higher demands are likely to impinge on people’s tendency to wear out and hence increase their tendency to become further distracted. Similarly, improved resources are likely to increase one’s extent of work engagement and hence improve one’s drive to complete work in spite of feeling ill. The link between job demands, resources and performance are well known (Bakker et al., 2004). One mechanism for this link resides in the notion that resources and demands trigger in decisions people take to action-out specific behaviors. For example, Gordon et al. (2015) demonstrated that demands and resources triggered decision making processes that influenced their state of engagement on task and contextual performance. In addition, their study also reported results implying that work demands trigger intuitive decision making toward a state of performance which is moderated by the extent of engagement. Given that presenteeism is also represented through a decision-making process (Aronsson and Gustafsson, 2005) of choosing willingly to attend, or otherwise, work while sick (Ruhle and Breitsohl, 2023), implies that integrating health-related productivity outcomes with the JD-R model is meaningful. After all, it has also been suggested that presenteeism may serve a therapeutic purpose (Karanika-Murray and Biron, 2020) thus implying similar mechanisms on good performance evaluations (Wang et al., 2022). In addition, Jenny et al. (2020) presented results that actually reveal the differential competing effects as a function of the interactions between resources and demands on productivity loss and gains and suggest the JD-R model as a strong conceptual framework to assess the interplay between demands and resources on eventual outcomes. Hence the choice of the JD-R for this purpose is warranted. Our study specifically assessed work-to-family conflict as a potential demand and staff support as a potential resource in line with two meta-analyses (Kossek et al., 2011; Vaziri et al., 2022). Therefore, the third research question investigated in this study is:

### Do the two SPS-6 factors provide theoretically meaningful relationships in a hypothesized model that explores employees’ occupational well-being?

**Figure 1:**
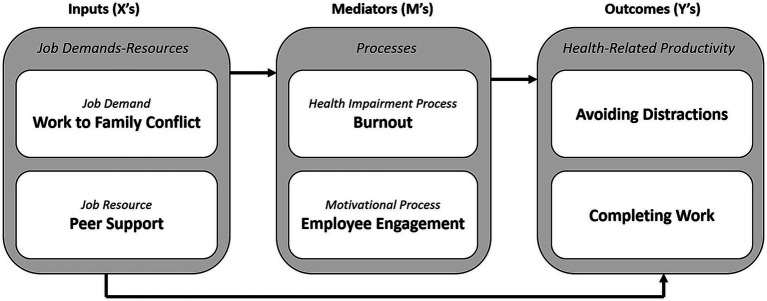
Conceptual model (JD-R) with SPS-6 factors.

The relationships among the variables in the JD-R model are illustrated in Figure 1.

## Method

### Participants

Our cross-sectional study targeted employees working in a multinational IT company with headquarters based in Poland who were fluent in the English language, had family responsibilities and were not remote workers. An email containing a weblink to the online questionnaire was sent to all the employees through the HR department, inviting them to participate willingly in our study. The respondents were not requested to provide personal identifiable information, while anonymity and confidentiality were guaranteed to reduce social desirability effects. Furthermore, to reduce confirmation bias, the respondents were instructed that there were no right or wrong answers in the statements provided, and to be honest as much as possible in their responses.

The sample size required for this study was determined by using the *a-priori* sample size calculator for structural equation models (Soper, 2023). After the following input parameters were specified – an anticipated effect size of 0.30 (medium effect), a probability level of 5%, a desired statistical power of 80%, 6 latent variables and 25 observed variables – the recommended minimum sample size required was 161. To be able to detect small effect sizes, the recommended sample size would inflate to 1,713. Between the 30th November and 12th December of 2021, we managed to secure complete responses from 268 respondents. Complete response was ensured (via mandatory fields) given that the type of statistical software used (AMOS) does not handle missing data. The demographic characteristics of the sample (*n* = 268) were as follows: the majority of the respondents were male (54.9%), in possession of a Bachelor’s degree (44.4%) or a Master’s degree (29.5%), and had full-time employment (75.4%). Additionally, the mean age of the respondents was 38.0 years (SD = 11.5), with ages ranging from 18 to 65 years.

### Measures

The questionnaire consisted of 29 items. The first four items requested demographic information from the participants while the remaining 25 items consisted of Likert-type items pertaining from established scale pertaining to the following constructs:

The Work-to-Family Conflict scale (Matthews et al., 2010) comprises three items. An example item is: “I am often so emotionally drained when I get home from work that it prevents me from contributing to my family.” Higher scores reflect higher work-to-family conflict. The mean score was 2.59 (SD = 0.99) while Cronbach’s alpha coefficient was 0.79.

The Peer Support items were taken from the revised ‘Health and Safety Executive’s Management Standards Indicator Tool’ (Cassar et al., 2020a). An example item is “I get all the help and support I need from work colleagues.” Higher scores are indicative of higher peer support. The mean score was 3.59 (SD = 0.07) while the Cronbach alpha coefficient was 0.92.

The Burnout scale (Malach-Pines, 2005) consists of 10 items and higher scores reflect higher burnout. An example item is: “When you think about your overall work, how often do you feel depressed?.” The mean score was 2.93 (SD = 1.09) while Cronbach’s alpha coefficient was 0.93.

The Employee Engagement scale comprises three items taken from the Work Engagement Scale (UWES-3; Schaufeli et al., 2019) and incorporates vigor, dedication and absorption. An example item is: “I am immersed in my work.” Higher scores indicate higher employee engagement. The mean score was 3.13 (SD = 1.04) while Cronbach’s alpha coefficient was 0.81.

Health-related productivity was assessed via Koopman et al.’s (2002) SPS-6. Three items pertain to Completing Work (e.g., “Despite my health problem, I felt energetic enough to complete all my work”) such that higher scores reflect a higher ability to accomplish work despite health problems. The remaining three items pertain to ‘Avoiding Distractions’ (e.g., “My health problem distracted me from taking pleasure in my work”). In line with Koopman et al. (2002), the measures generated from these three items were reverse-scored such that higher scores reflect a higher ability to concentrate on work despite health problems. The mean scores for Completing Work and Avoiding Distractions were 3.26 (SD = 0.96) and 3.00 (SD = 1.01) respectively while the Cronbach alpha coefficients were 0.84 and 0.80, respectively.

For all the construct items, the respondents were asked to rate their level of agreement with response options ranging from 1 = strongly disagree to 5 = strongly agree, with the exception of Burnout whose response options ranged from 1 = never to 7 = always.

### Data analysis procedure

To answer RQ1 and RQ2, we constructed two CFA models in AMOS (Version 27) containing the 25 observed variables. The first CFA model contained 6 latent variables (work-to-family conflict, peer support, burnout, and the two dimensions of presenteeism – completing work and avoiding distractions) while the second CFA model included the second-order factor of presenteeism with completing work and avoiding distractions as its underlying sub-constructs. To assess goodness of fit, we generated and interpreted the Chi-square statistic to the respective degrees of freedom (χ2/df), the Confirmatory Fit Index (CFI), the Root Mean Square Error of Approximation (RMSEA) and the Standardized Root Mean Square Residual (SRMR). The following thresholds recommended by Hu and Bentler (1999) were adopted:, a *χ*^2^/*df* ≤ 3 a CFI ≥ 0.95, an RMSEA ≤0.05 and an SRMR ≤0.09. The Chi-square difference test and the Expected Cross-Validation index (ECVI) were used to determine which of competing models represented the best fit. The ECVI has “no determined appropriate range of values” but the model with the smallest ECVI value exhibits the greatest potential for replication” (Byrne, 2001, p. 86).

To demonstrate evidence of the convergent validity of the SPS-6 measures, the CR index must be at least 0.7 (note: reliability is a prerequisite of validity) and the AVE must be at least 0.5 (Hair et al., 2010). To demonstrate evidence of discriminant validity, the MSV must be smaller than the AVE, while the square root of the AVE value must be greater than the absolute value of the inter-construct correlation/s (Hair et al., 2010).

To assess the concurrent validity of the SPS-6 measures (RQ3), we used Structural Equation Modelling (SEM). For the JD-R model, we specified *X*_1_ = Work-to-Family Conflict (job demand) and *X*_2_ = Peer Support (job resource) as exogenous variables, *M*_1_ = Burnout and *M*_2_ = Employee Engagement as mediators, and *Y*_1_ = Completing Work and *Y*_2_ = Avoiding Distractions as outcomes. After assessing the goodness-of-fit of this structural model and conducting modifications (by correlating error terms where necessary), we tested for direct and indirect effects. For the direct effects, the statistical significance of regression coefficients for specific paths in the model were interpreted; here a significant *t*-statistic signifies a significant direct effect. To examine specific indirect effects in mediation analysis, the bias-corrected percentile method was used with 5,000 bootstrap samples and 95% confidence in AMOS (Version 27). A significant bootstrap value of *p* for a user-defined estimand implied that the specific indirect effect was significantly different from zero. To determine the extent of the mediation (no mediation, complementary partial mediation, competitive partial mediation or full mediation), we followed guidelines by Zhao et al. (2010). In other words, no mediation occurs when the indirect effect (a × b) is not significant. Full mediation occurs when only the indirect effect is significant but the direct effect (c’) is not significant. Finally, partial mediation occurs when both the direct and indirect effects are significant; if they point in the same direction (i.e., the sign of a × b × c’ is positive), the partial mediation is complementary but if they point in opposite directions (i.e., the sign of a × b × c’ is negative), then the partial mediation is competitive.

## Results

### Factor structure of the SPS-6 measures

The first CFA model (Model 1) investigated included 6 latent variables (namely, work-to-family conflict, peer support, burnout, employee engagement, completing work and avoiding work) and 25 indicator variables. The initial model produced a reasonably acceptable fit to the data (*χ*^2^/*df* = 2.05, CFI = 0.93, RMSEA = 0.06, SRMR = 0.05). To identify areas of misfit, we inspected modification indices for covariances and went on to correlate error terms associated with the burnout indicators, starting with the error terms of B1 and B2 (i.e., e7 and e8), followed by those of B3 and B4 (i.e., e9 and e10), B9 and B10 (i.e., e15 and e 16), B3 and B5 (i.e., e9 and e11) and B4 and B5 (i.e., e10 and e11). Table 1 provides a summary of statistical output regarding model 1 improvement, re-specification and comparison from Model 1A to Model 1F.

**Table 1:** CFA model improvement, re-specification and comparison (Model 1).

	Modification		Goodness-of-fit indices					
Model	Error terms	M.I. (Par. Δ)	*χ^2^* *(df)*	*Δ χ^2^*	*χ^2^*/*df*	CFI	RMSEA	SRMR
1A	–	– (−)	561.625 (260)	–	2.045	0.928	0.063	0.050
1B	e7↔e8	25.516 (0.266)	505.127 (259)	56.498^**^	1.950	0.934	0.060	0.049
1C	e9↔e10	23.931 (0.226)	478.318 (258)	26.809^**^	1.854	0.941	0.057	0.048
1D	e15↔e16	14.261 (0.313)	463.339 (257)	14.979^**^	1.803	0.945	0.055	0.048
1E	e9↔e11	9.050 (0.112)	453.290 (256)	10.049^*^	1.771	0.950	0.053	0.047
1F	e10↔e11	13.114 (0.146)	433.773 (255)	19.517^**^	1.701	0.952	0.050	0.046

M.I., modification index, ^*^p ≤ 0.01, ^**^p ≤ 0.001.

**Figure 2:**
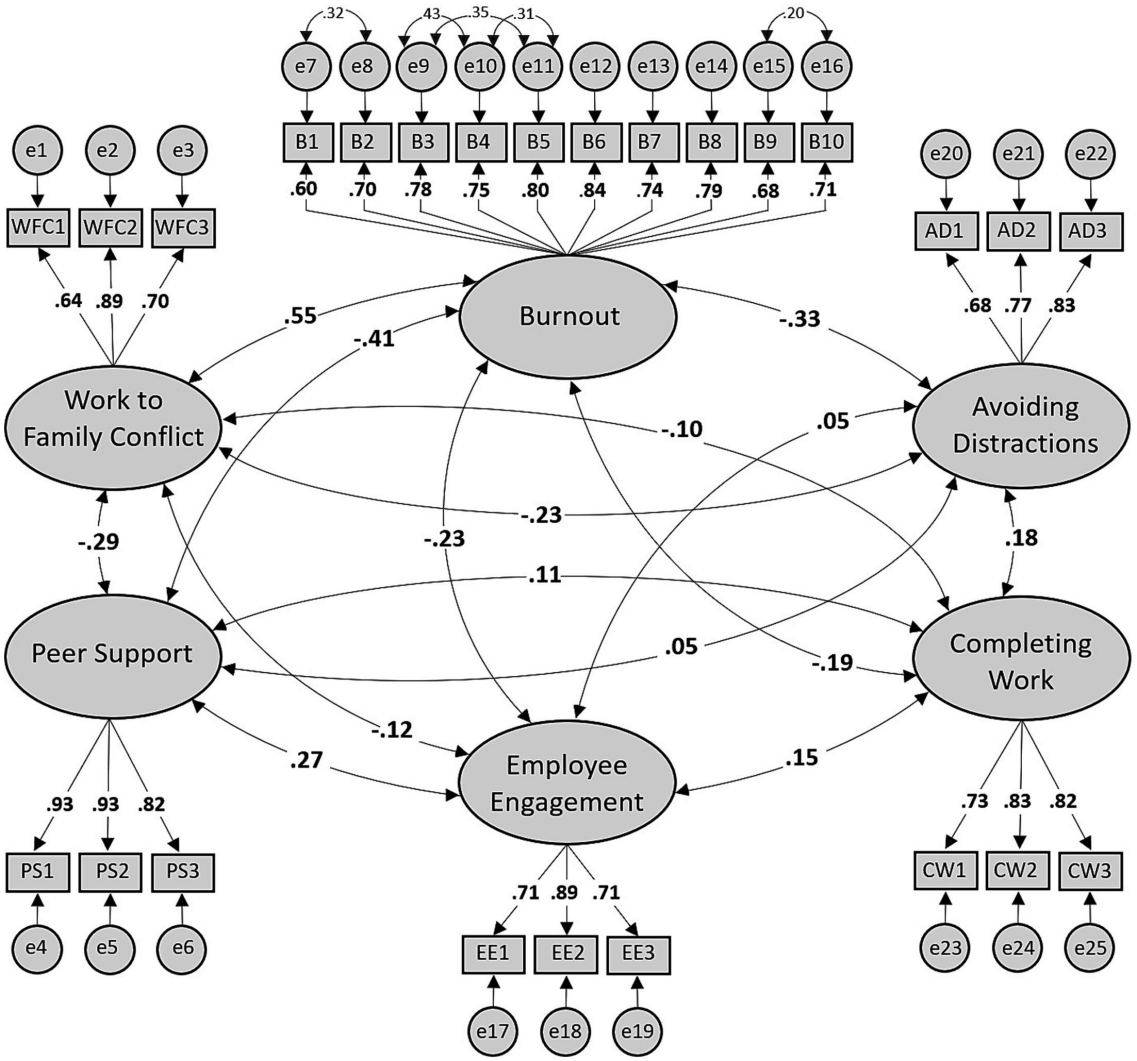
CFA model with standardized loadings (Model 1F).

Table 1 shows that each re-specification produced incremental improvement in model fit estimations as indicated by the Chi-square difference test and other goodness of fit measures such that the final model (Model 1f) produced a good fit to the data (*χ*^2^/*df* = 1.70, CFI = 0.95, RMSEA = 0.05, SRMR = 0.05, ECVI = 2.15), according to guidelines by Hu and Bentler (1999). Furthermore, the ECVI dropped from 2.48 for Model 1A to 2.15 for Model 1F (Byrne, 2001). Figure 2 provides the standardized factor loadings for Model 1F.

Figure 2 shows that the six factors were stable since their loadings all exceeded 0.6 while their average factor loadings clearly exceeded 0.7 (Mac Callum et al., 2001), implying that more of the variance in the factors is explained variance than error variance (Hair et al., 2010). We then proceeded to assess the convergent and discriminant validity of the study measures (see Table 2).

**Table 2:** Inter-factor correlations and convergent/discriminant validity measures with two SPS-6 first-order factors (Model 1).

Construct	CR	AVE	MSV	WFC	PS	B	EE	CW	AD
WFC	0.792	0.564	0.304	**0.751**	−0.294**	0.551**	−0.124*	*−0.096*	−0.232**
PS	0.926	0.808	0.166		**0.899**	−0.408**	0.271**	*0.108*	*0.051*
B	0.924	0.550	0.304			**0.742**	−0.230**	−0.190**	−0.330**
EE	0.816	0.600	0.073				**0.774**	0.146*	*0.051*
CW	0.838	0.634	0.036					**0.796**	0.183**
AD	0.804	0.580	0.109						**0.761**

WFC, work-to-family conflict; PS, peer support; B, burnout; EE, employee engagement; CW, completing work; AD, avoiding distractions; CR, composite reliability; AVE, average variance extracted; MSV, maximum shared variance, the Square Root of AVE is presented in bold while inter-factor correlations are presented in italics; **p ≤ 0.01 (two-tailed), *p ≤ 0.05 (two-tailed).

Table 2 shows that all the six construct measures utilized in this study did not have any validity concerns. In fact: (a) with respect to reliability, which is a prerequisite for validity, the CR indices are higher than 0.7; (b) with respect to convergent validity, the AVEs exceeds 0.5; and (c) with respect to discriminant validity, the MSV’s are smaller than their respective AVEs, while the square root of the AVEs of the factors exceed the absolute values of the correlations with the other respective factors.

It is also worth noting that the inter-factor correlations are all in the expected theoretical direction Additionally, the correlation between the two SPS-6 factors is weak (*r* = 0.18), making it quite unreasonable to suppose that the second-order factor of health-related productivity may be producing the associations among the SPS-6 first-order factors (Brown, 2015).

Therefore, with respect to RQ1, this study re-confirms that the SPS-6 indicators adequately capture the underlying constructs (Brown, 2015) of ‘completing work’ and ‘avoiding distractions’. Furthermore, these two SPS-6 measures demonstrate adequate convergent and discriminant validity.

### The second-order factor of health-related productivity

**Figure 3:**
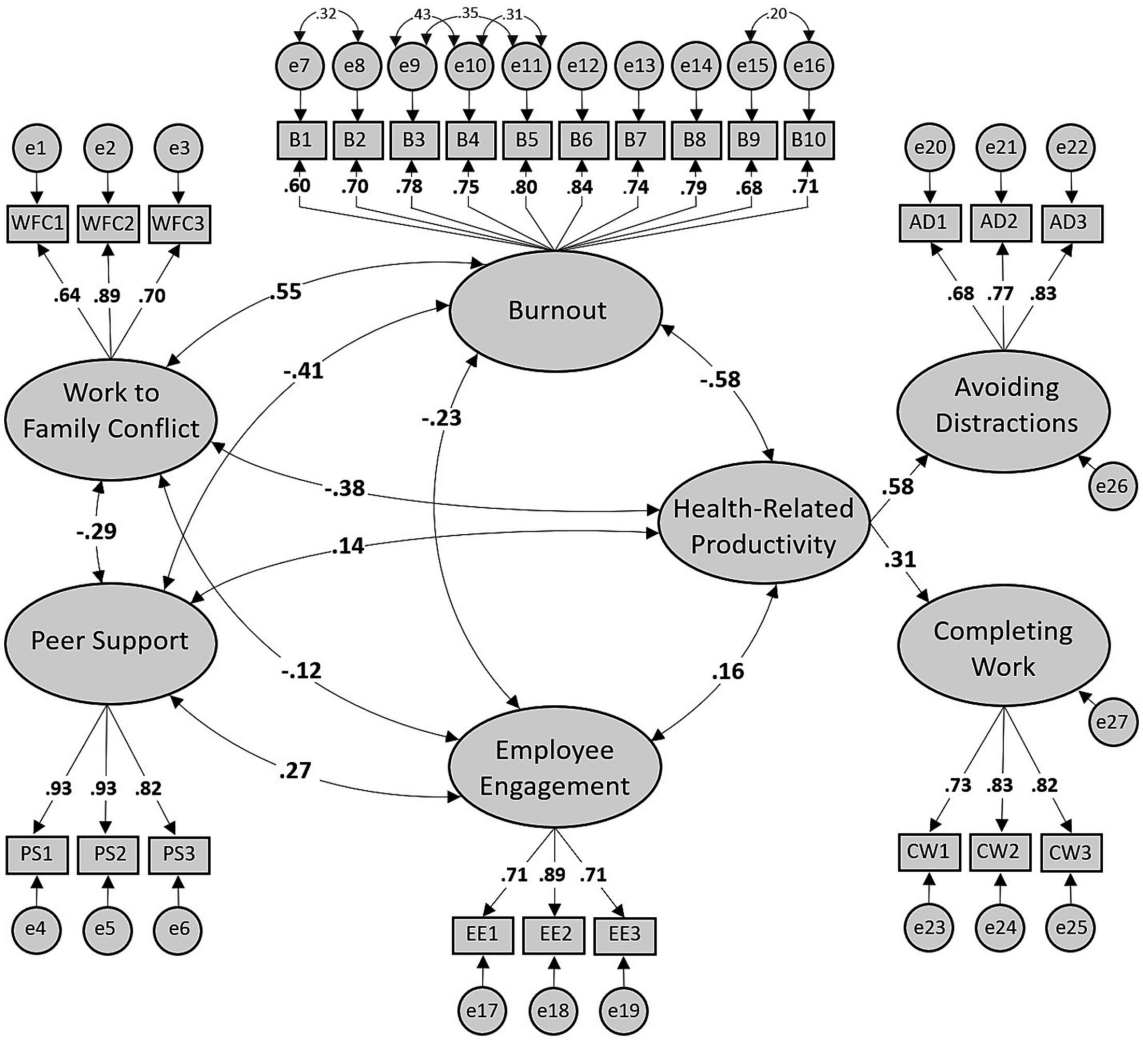
CFA model with standardized loadings (Model 2).

The second CFA model (Model 2) contained the same latent variables and indicator variables like Model 1 but included another latent variable to represent the second order factor of health-related productivity, with completing work and avoiding distractions as its underlying sub-constructs. As expected, CFA Model 2, which is exhibited in Figure 3, produced similar fit indices to Model 1. In fact, the goodness-of-fit indices (*χ*^2^/*df* = 1.70, CFI = 0.95, RMSEA = 0.05, SRMR = 0.05) were good (Hu and Bentler, 1999).

However, the factor loadings from the second-order factor of health-related productivity to completing work and avoiding distractions were 0.31 and 0.58, respectively. One cannot consider this second-order factor to be stable since both these loadings are below 0.6 (Mac Callum et al., 2001). Furthermore, their average factor loading is 0.45 which does not exceed 0.7, implying that more of the variance in the factors is error variance than explained variance (Hair et al., 2010). We then proceeded to assess the convergent and discriminant validity of the second-order factor scores (see Table 3).

**Table 3:** Inter-factor correlations and convergent/discriminant validity measures with second-order factor of health-related productivity (Model 2).

Construct	CR	AVE	MSV	WFC	PS	*B*	EE	HRP
WFC	0.792	0.564	0.304	**0.751**	−0.294**	0.551**	−0.124*	−0.382**
PS	0.926	0.808	0.166		**0.899**	−0.408**	0.270**	0.136*
B	0.924	0.550	0.304			**0.742**	−0.230**	−0.575**
EE	0.816	0.600	0.073				**0.775**	0.157*
HRP	0.345	0.226	0.330					**0.467**

WFC, work-to-family conflict; PS, peer support; B, burnout; EE, employee engagement; CW, completing work; HRP, health-related productivity; CR, composite reliability; AVE, average variance extracted; MSV, maximum shared variance, the Square Root of AVE is presented in bold while inter-factor correlations are presented in italics; **p ≤ 0.01 (two-tailed), *p ≤ 0.05 (two-tailed).

Table 3 shows that the second-order factor of health-related productivity has convergent and discriminant validity issues. This is because its CR is below 0.7, its AVE is less than 0.5, its MSV is larger than the AVE, and the square root of its AVE is smaller than the absolute value of the correlation with at least another factor, namely burnout (Hair et al., 2010).

Therefore, with respect to RQ2, an examination of factor loadings, convergent validity, and discriminant validity revealed that the second-order factor of health-related productivity did not produce sufficient associations with its sub-constructs, namely the first-order dimensions of the SPS-6 (Brown, 2015). This implies that the SPS-6 is better represented by the two separate dimensions of completing work and avoiding distraction than an aggregated dimension of health-related productivity.

### Investigating direct and indirect effects.

A structural model in line with the conceptual framework highlighted in Figure 1 was constructed. Although the Chi-square statistic was statistically significant [χ^2^(257) = 440.08, *p* < 0.01] the other goodness-of-fit indices suggested a reasonably good fit (*χ*^2^/*df* = 1.71, CFI = 0.95, RMSEA = 0.05, SRMR = 0.05). Table 4 provides a summary of SEM output for the direct effects.

**Table 4:** Parameter estimates for direct effects.

Paths	Unstandardized		Standardized	Critical ratio	Value of *p*
	Estimate	S.E.	Estimate		
WFC➔B	0.382	0.067	0.473	5.704	<0.001
WFC➔EE	−0.070	0.085	−0.061	−0.826	0.409
PS➔B	−0.158	0.037	−0.272	−4.268	<0.001
PS➔EE	0.212	0.060	0.256	3.529	<0.001
WFC➔CW	0.018	0.103	0.015	0.171	0.864
PS➔CW	0.010	0.065	0.012	0.874	0.874
WFC➔AD	−0.107	0.118	−0.081	0.914	0.361
PS➔AD	−0.104	0.074	−0.109	−1.409	0.159
B➔CW	−0.249	0.132	−0.174	−1.880	0.060
B➔AD	−0.540	0.157	−0.330	−3.444	<0.001
EE➔CW	0.106	0.075	0.106	1.416	0.157
EE➔AD	−0.001	0.084	−0.001	−0.017	0.986

WFC, work-to-family conflict; PS, peer support; B, burnout; EE, employee engagement; CW, completing work; AD, avoiding distractions.

**Figure 4:**
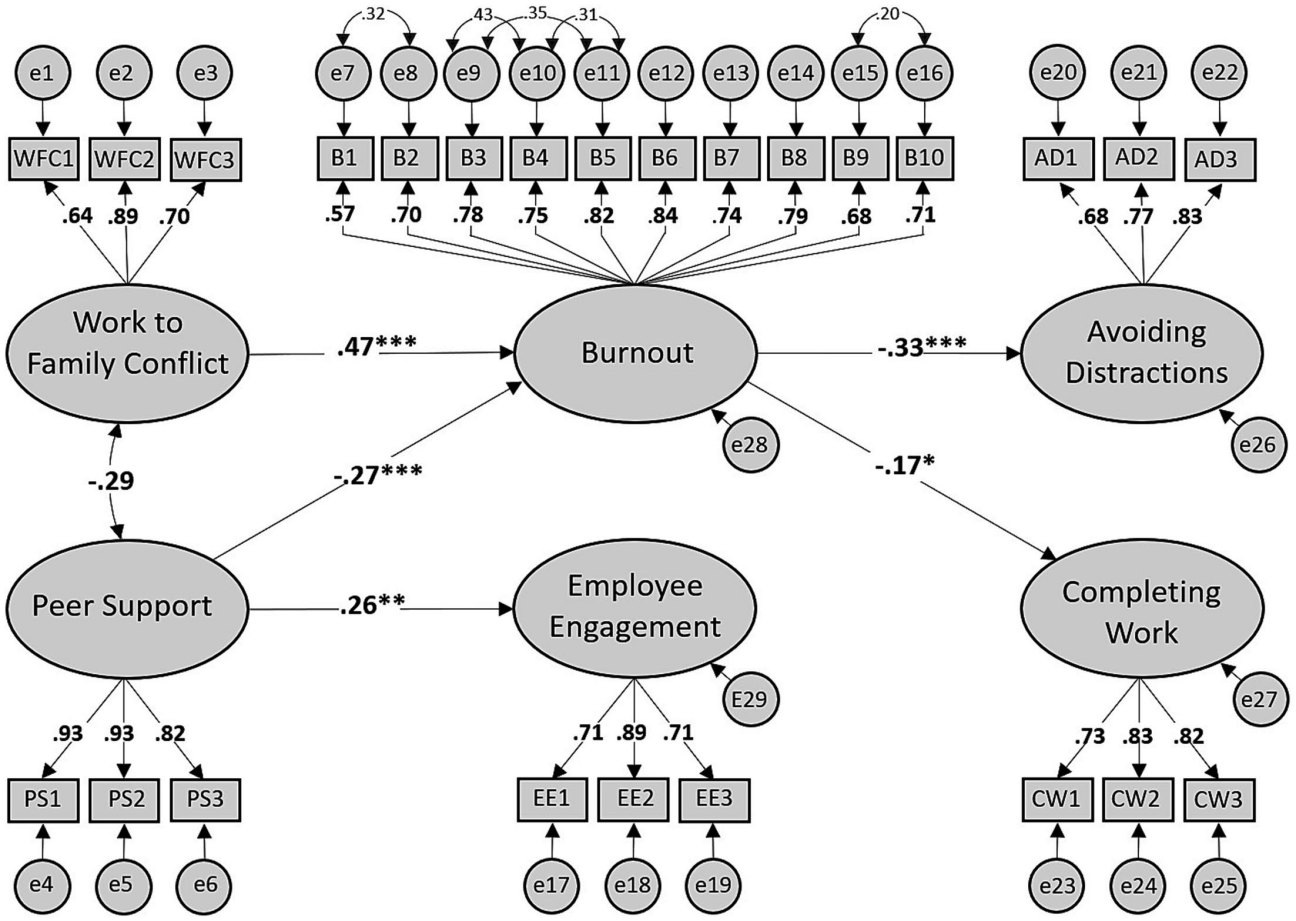
Direct effects path diagram with standardized loadings for significant paths (Model 3). *p ≤ 0.10, **p ≤ 0.05, ***p ≤ 0.01.

Table 4 shows that from the 12 direct paths investigated, the regression weights of four paths emerged as statistically significant. These were the paths from (a) work-to-family conflict to burnout (*β* = 0.47, *p* < 0.01), (b) peer support to burnout (*β* = −0.27, p < 0.01) (c) peer support to employee engagement (*β* = 0.26, *p* < 0.01), and (d) burnout to avoiding distractions (*β* = 0.33, *p* < 0.01). The regression weight of the path from burnout to completing work (*β* = −0.17, *p* = 0.06) was marginally significant. Figure 4 illustrates the structural model with standardized loadings for the significant direct effects. The non-significant paths and their standardized regression weights are not shown to avoid clutter. Given non-significant direct effects from the inputs to the outcomes and from employee engagement as mediator to the outcomes, burnout emerges as the only contestant that produced full mediation influences.

Table 5 provides a summary of SEM output for the specific indirect effects.

**Table 5:** Indirect effects (mediation).

Hypothesis	Estimate^a^	Bootstrap 95% CI		Sig. (two-tailed)	Extent of mediation
		Lower	Upper		
WFC➔B➔CW	−0.095	−0.240	0.014	0.089	No mediation
WFC➔B➔AD	−0.206	−0.411	−0.079	0.001	Full mediation
WFC➔EE➔CW	−0.007	−0.058	0.008	0.300	No mediation
WFC➔EE➔AD	0.000	−0.020	0.026	0.915	No mediation
PS➔B➔CW	0.039	−0.003	0.105	0.064	No mediation
PS➔B➔AD	0.086	0.028	0.183	0.001	Full mediation
PS➔EE➔CW	0.023	−0.009	0.078	0.180	No mediation
PS➔EE➔AW	0.000	−0.035	0.045	0.999	No mediation

^a^Standardized regression weight for indirect effect; WFC, work to family conflict; PS, peer support; B, burnout; EE, employee engagement; CW, completing work; AD, avoiding distractions.

Table 5 revealed that burnout fully mediated the relationships between (a) work-to-family conflict and avoiding distractions to work (*β* = −0.21, *p* < 0.01, 95% CI = [−0.41, −0.08]), and (b) peer support and avoiding distractions to work (*β* = 0.09, *p* < 0.01, 95% CI = [0.03, 0.18]). The confidence intervals of the remaining indirect effects contained a zero, implying no mediation.

## Discussion

### Summary of key findings

Presenteeism is an important construct in the general work well-being literature which requires that researchers measure it adequately in order to better understand the underlying mechanics of both its antecedents as well as outcomes (Lohaus and Habermann, 2019). This is also in line with a need for more measures to be aligned to theory and for theory to be embedded in strong explanatory frameworks (Colquitt and Zapata-Phelan, 2007). Part of this approach is to strengthen the alignment between construct and measurement (Suddaby, 2010) and presenteeism is no exception (Johns, 2010; Hutting et al., 2014; Ruhle et al., 2020).

This study sought to contribute in this direction by examining a well-sought self-report measure in the field of presenteeism, namely, the SPS 6-item measure (Koopman et al., 2002), by revisiting its psychometric properties (Gosselin et al., 2013; Lohaus and Habermann, 2019). The SPS-6 has been more recently applied to assess health-related productivity loss as a consequence of attending work while sick. In particular, three objectives were set for this study: first to examine the convergent and discriminant validity of its construct measures; second to examine its acclaimed single aggregated measure (the total SPS-6 score); and third to examine its explanatory role in the wider well-being domain by using the JD-R model as a framework.

In summary, this cross-sectional study revealed that first, the two SPS-6 dimensions - completing work and avoiding distractions - demonstrated good convergent and discriminant validity. Overall, results of this study build toward enhanced knowledge of presenteeism and use of the SPS-6 measures (Koopman et al., 2002) as health-related productivity outcomes. Results demonstrate that the measure is best represented by two dimensions, one representing the tendency for people to engage actively at work while feeling sick or ill (Completing Work) and the other representing the tendency for people to feel distracted at work because of their ill-health (Avoiding Distractions). Indeed, our results strengthened this conviction given the correlation between the two dimensions was weak, suggesting that the two dimensions are unique facets. In addition, convergent and discriminant validity issues for the second-order factor scores enhanced this informed opinion. Indeed, the results suggested that there was no substantial correlation that makes it reasonable to suppose a second-order factor may be producing the associations among the first-order factors (Brown, 2015). This runs counter to the claim by Koopman et al. (2002) and others that the two factor scores should be summated to produce a total SPS-6 score. This is an important finding in itself as all other versions of the measure have simply added the scores of both dimensions. In some cases, researchers provided scores to reflect ‘loss’ (e.g., Hutting et al., 2014; Li et al., 2019) while others provided scores to reflect higher performance while feeling sick at work (e.g., Koopman et al., 2002; Cicolini et al., 2016). Our findings suggest therefore that while both dimensions reside within the same construct, they exert a life of their own. Current literature in presenteeism has indeed considered the multifaceted nature of the phenomenon (e.g., Patel et al., 2023), and it may perhaps be interesting to consider the extent that the health-related performance outcomes are also multifaceted.

Second, our results suggest that the items for both dimensions typically signify different consequences as a function of presenteeism and future studies may wish to expand to dissect the outcomes into productivity loss (avoiding distractions) and productivity gain or maintenance (completing work). These two dimensions are also in line with more current conceptualizations of presenteeism that argue that rather than conceiving it as one single phenomenon, presenteeism may have both its advantages and disadvantages to work (Johns, 2010; Karanika-Murray and Biron, 2020; Biron et al., 2022) because of its potentially dual nature. In addition, our initial workings with both dimensions treated separately suggested that while work resources were more likely to be associated with completing work (through engagement), work demands were more likely to be associated with avoiding distractions (through burnout). This pattern of results further suggests that the two components of presenteeism are likely to be predicted to a degree by the proportionality of demands to resources with a slighter strong effect through burnout. This is in line with the literature which may have come across as contradictory to each other with some studies arguing a positive relationship between presenteeism and negative states of wellness (Johns, 2010; Garrow, 2016) and others finding a more negative or no relationship with such states of wellness (Lu et al., 2013). This is also much in accordance to a number of calls by researchers such as Miraglia and Johns (2016) whose meta-analysis found that a dual process model (positive and negative elements of presenteeism) was better at clarifying how job demands and resources elicit presenteeism via both health impairments and motivational pathways and this variation was stronger in presenteeism than absenteeism. Our findings also echo conceptual and theoretical positions asserted by other researchers (Ruhle et al., 2020; Biron et al., 2022). It is important to highlight that completing work requires further investigation as a positive outcome even though the item-semantic construction of the measure and the results point into that direction (negatively related to burnout). Indeed, one may contemplate the relationship between completing work and other positive oriented constructs such as resilience (e.g., McCray and Joseph-Richard, 2020).

### Theoretical and practical considerations when using the SPS-6

Our findings present a number of theoretical and practical considerations for researchers using the SPS-6. Theoretically, the SPS-6 should be seen as possessing both a potentially negative component which can be conceptualized as productivity loss as it indicates the tendencies for people to feel worse by going to work in a state of subjective sickness. However, it also has an adaptive aspect which has been overlooked (Karanika-Murray and Biron, 2020). The notion of completing one’s work in spite of feeling sick requires inclusion in more updated theoretical frameworks in order to detach it from the general tendency to assimilate it as a mere diagonal opposite of absenteeism (Gosselin et al., 2013). The use of stress-based models like the JD-R integrated within a decision-making process that determines people’s motivation to take specific courses of action may shed more light on how the balance between losing out on productivity versus maintaining adequate standards of performance are achieved (Gordon et al., 2015; Jenny et al., 2020). Attending work while being sick or ill is a choice that is potentially determined by a fine balance between resources and demands triggering different psychological states and potentially health-related productivity outcomes (Ruhle and Breitsohl, 2023). From a practical perspective, it implies that managing ‘presenteeism’ requires understanding the underlying reasons why people attend work and how their state of presenteeism is fueled (engagement versus burnout for instance) to produce health-related productivity outcomes. The fact that presenteeism means that people are working (as opposed to absenteeism) requires managers to ensure that such states are more beneficial and adaptive rather than disruptive and unproductive. Managers can ensure this by supporting employees with better personal and job resources and reducing the impacts of perceived demands.

### Limitations and avenues for further research

Of course, our study also has a number of limitations that should be addressed in further studies to improve its generalizability. First, our study is based on a single cross-sectional investigation. This means that the pathways determining the two components of presenteeism in the JD-R model are based on the theoretical notion that demands/resources impact engagement/burnout which then impact forms of health-related presenteeism outcomes. Further studies should explore the longitudinal nature of these claims including of how reverse causal pathways may also be possible. For example, the sense of completing work while sick may convey such a strong willingness to override ill-symptoms that people are intrinsically energized and engage better with their tasks. On the contrary, the more people attend work and feel having to compete between their work and their attendance to their ill-health may make them perceive simple challenges as more highly demanding contributing to further burnout. In addition, the role of burnout against engagement should be further examined to assess which of the two has more weight on different aspects of health-related productivity and hence understand whether merely improving resources is a better approach compared to effectively managing demands.

Second, our study focused on jobs that are traditionally completed in a physical environment with clear boundaries between work and non-work. In our case, although work was conducted during the Covid-pandemic, our sample was required to work from their place of work, and any possible additional symptoms such as the fear of getting sick were not controlled for (e.g., Kinman and Grant, 2021). We are also aware that new emerging forms of work also include working from home and this became more accentuated after the pandemic (Caligiuri et al., 2020). This has led some scholars to highlight the need to review the phenomenon of presenteeism in light of changes to how people work and new forms of work including hybrid forms (Breitsohl et al., 2023). We urge researchers to explore how presenteeism and more specifically different aspects of it are related, impacted or managed by emerging new forms of work.

Third, future research should examine a wider range of resources and demands. In our case we only examined one resource (peer support) and one demand (work-to-family conflict). This should help to provide a better understanding of the underlying mechanics of ‘presenteeism’ within established frameworks of well-being.

## Conclusion

Presenteeism is a phenomenon that merits attention because it has an impact on productivity, health and costs. In order to understand its mechanics better, one needs to ensure that its conceptualization is reflected in the tools used to measure it. Our contribution to the literature has been to revisit one of the most popular measures of presenteeism by looking more into its psychometric properties. Our line of results suggests that the SPS-6 (Koopman et al., 2002) is a good assessment but it would be more beneficial to use it to measure health-related productivity outcomes by using both dimensions separately and without reverse scoring because the two dimensions seem to denote different productivity outcomes in the light of presenteeism. In addition, doing so should provide better understanding about the phenomenon which has strong theoretical foundations and elicits more sound management applications.

## Data availability statement

The raw data supporting the conclusions of this article will be made available by the authors, without undue reservation.

## Ethics statement

The studies involving humans were approved by Faculty Research Ethics Committee, FEMA, University of Malta. The studies were conducted in accordance with the local legislation and institutional requirements. The participants provided their written informed consent to participate in this study.

## Author contributions

All authors listed have made a substantial, direct, and intellectual contribution to the work and approved it for publication.
